# The therapeutic value of SC66 in human renal cell carcinoma cells

**DOI:** 10.1038/s41419-020-2566-1

**Published:** 2020-05-11

**Authors:** Ming Xu, Yin Wang, Li-Na Zhou, Li-jun Xu, Zhi-chang Jin, Dong-rong Yang, Min-bin Chen, Jin Zhu

**Affiliations:** 1 Department of Urology, The Second Affiliated Hospital of Soochow University, Suzhou, China; 2 Institute of Neuroscience, Soochow University, Suzhou, China; 3 Department of Radiotherapy and Oncology, Affiliated Kunshan Hospital of Jiangsu University, Suzhou, China; 4 Department of Urology, Ningbo Urology Nephrology Hospital, Ningbo, China

**Keywords:** Targeted therapies, Targeted therapies

## Abstract

The PI3K-AKT-mTOR cascade is required for renal cell carcinoma (RCC) progression. SC66 is novel AKT inhibitor. We found that SC66 inhibited viability, proliferation, migration and invasion of RCC cell lines (786-O and A498) and patient-derived primary RCC cells. Although SC66blocked AKT-mTORC1/2 activation in RCC cells, it remained cytotoxic in AKT-inhibited/-silenced RCC cells. In RCC cells, SC66 cytotoxicity appears to occur via reactive oxygen species (ROS) production, sphingosine kinase 1inhibition, ceramide accumulation and JNK activation, independent of AKT inhibition. The ROS scavenger N-acetylcysteine, the JNK inhibitor (JNKi) and the anti-ceramide sphingolipid sphingosine-1-phosphate all attenuated SC66-induced cytotoxicity in 786-O cells. In vivo, oral administration of SC66 potently inhibited subcutaneous 786-O xenograft growth in SCID mice. AKT-mTOR inhibition, SphK1 inhibition, ceramide accumulation and JNK activation were detected in SC66-treated 786-O xenograft tumors, indicating that SC66 inhibits RCC cell progression through AKT-dependent and AKT-independent mechanisms.

## Introduction

Renal cell carcinoma (RCC) is the most common type of renal malignancy^1^. Nephroureterectomy of early-stage RCC is the only possible curable treatment option^1^. However, RCC is more often diagnosed at an advanced stage, with 25% of patients developing local invasion and systematic metastasis resulting in a poor prognosis^1^. The PI3K-AKT-mTOR signaling pathway is frequently upregulated in RCC due to mechanisms that include PTEN mutation/depletion, PI3KCA mutation, and sustained activation of receptor tyrosine kinases (RTKs)^2–5^. Constitutive activation of this cascade is necessary for RCC cell proliferation, survival, migration, and metastasis, and also angiogenesis and resistance to anti-tumor treatments^2,3,6,7^. Molecularly-targeted agents are currently being utilized for the treatment of certain RCC patients, including the mTORC1 inhibitors Temsirolimus and everolimus, which are approved by the FDA for the treatment of advanced RCC^2,3,6,7^.

Our group has previously shown that targeted inhibition of the PI3K-AKT-mTOR pathway is a valid treatment strategy in the management of RCC^8–10^. SF2523, a PI3K-AKT and bromodomain-containing protein 4 (BRD4) dual inhibitor, was found to potently inhibit RCC cell growth in vitro and in vivo^8^. Similarly, the AKT-mTORC1/2 inhibitor WYE-687 inhibited cell growth of human RCC cells^9^. In addition, we identified that microRNA-302c inhibited RCC cell proliferation by targeting Grb2-associated binding 2 (Gab2)-AKT signaling^10^.

Jo et al., developed SC66^11^, a novel allosteric AKT inhibitor that exerted a dual-inhibitory mechanism by inducing AKT ubiquitination and interfering with AKT pleckstrin homology (PH) domain binding to PIP3^11^. The study by Cusimano et al., demonstrated that AKT inhibition by SC66 induced significant cytotoxic effects in hepatocellular carcinoma (HCC) cells^12^. In this study, we demonstrate that in addition to AKT-dependent RCC cell inhibition, SC66 inhibits RCC cell progression via AKT-independent mechanisms.

## Materials and methods

### Reagents and chemicals

SC66, MK-2206, and LY294002 were purchased from MCE Chemicals (Shanghai, China). N-acetylcysteine (NAC), PD98059, U0126, sphingosine-1-phosphate (S1P) and the JNK inhibitor (JNKi) were provided by Sigma-Aldrich (St. Louis, Mo). The broad caspase inhibitor z-VAD-cho and the caspase-3 inhibitor z-DEVD-cho were obtained from Enzo Life Sciences (Shanghai, China). Antibodies for phosphorylated (“p”)-AKT (Ser-473) (#9271), AKT1/2 (#9272), p-S6K1 (#9234), S6K1 (9202), p-Erk1/2 (#9101), Erk1/2 (#9102), p-JNK (#9255), JNK1/2 (#9252), SphK1 (#12071), cleaved-caspase-3 (#9664), cleaved-caspase-9 (#20750), cleaved-poly (ADP-ribose) polymerase (PARP) (#5625), Bcl-2 (#15707), and *β*-tubulin (#15115) were purchased from Cell Signaling Tech (Beverly, MA).

### Cell culture

The established RCC cells (786-O and A489 lines) and immortalized HK-2 tubule epithelial cells^13,14^ were cultured using the previous protocol^10,15^. Cells were routinely subjected to mycoplasma and microbial contamination examination. STR profiling, population doubling time, and morphology were routinely checked every 3–4 months to confirm the genotype. The primary human RCC cells, derived from three primary RCC patients (“RCC1/2/3”), as well as the primary human renal epithelial cells (“Ren-Epi”) were cultured in the described medium^8,9^. The written-informed consent was obtained from each enrolled patient. All investigations were conducted according to the principles expressed in the Declaration of Helsinki. Experiments and protocols were approved by the Ethics Review Board of Soochow University (Suzhou, China).

### Methylthiazol tetrazolium (MTT) assay

Cells were seeded onto the 96-well tissue culture plates (3 × 10^3^ cells per well). Following treatment, cell viability was assessed by the MTT assay. MTT OD was recorded at 490 nm.

### Soft agar colony formation assay

Cells were seeded onto the 10-cm tissue culture dishes (1 × 10^4^ cells per dish), treated with SC66 every two days for five rounds. Afterwards, the number of viable 786-O colonies were counted.

### BrdU assay

Cells were seeded onto the six-well tissue culture plates (1 × 10^5^ cells per well). Following treatment, cells were incubated with BrdU (10 μM, Cell Signaling Tech) for 8 h and then fixed. BrdU incorporation was determined in the ELISA format. BrdU OD at 405 nm was recorded.

### EdU assay of cell proliferation

Cells were seeded onto the six-well tissue culture plates (1 × 10^5^ cells per well). The EdU (5-ethynyl-20-deoxyuridine) Apollo-488 In Vitro Imaging Kit (Ribo-Bio, Guangzhou, China) was utilized to quantify cell proliferation. Following treatment EdU (2.5 μM) was added to RCC/epithelial cells for 6 h. Cell nuclei were stained with Hoechst-33342 for 5 min, visualized under a fluorescent microscope (Leica). We counted at least 400 cells of six random views to calculate EdU ratio for each treatment.

### In vitro cell migration and invasion assays

As described^16,17^ RCC cells (4 × 10^4^ cells of each condition in 200 μL serum-free medium) were initially seeded onto the upper surfaces of “Transwell” chambers. The lower compartments were always filled with complete medium (containing 10% FBS). Following 24 h incubation, the migrated cells on the lower surface were fixed, stained and counted. Matrigel (Sigma) was added in the chamber surfaces when analyzing cell invasion.

### Caspase activity assay

Assaying of caspase-3/-9 activity was described previously^18^. Twenty μg of cytosolic extracts of each treatment were added to the caspase assay buffer^18^ with the caspase-3 substrate or the caspase-9 substrate^18^. Release of 7-amido-4-(trifluoromethyl)-coumarin (AFC) was quantified by using a Fluoroskan system^18^. AFC optic density (OD) was recorded.

### Annexin V FACS assay

As reported^18^, cells with the indicated treatment were washed and incubated with Annexin V-FITC (10 μg/mL) and propidium iodide (PI, 10 μg/mL) (Invitrogen), and detected by fluorescence-activated cell sorting (FACS) using a Becton-Dickinson machine. Annexin V-positive cells were labeled as the apoptotic cells.

### TUNEL assay

Cells were seeded onto the six-well tissue culture plates (1 × 10^5^ cells per well). Following treatment, cells were incubated with TUNEL (Invitrogen, 10 μM) for 3 h. Cell nuclei were stained with Hoechst-33342 for 5 min, visualized under a fluorescent microscope (Leica). For each treatment, we counted at least 400 cells of six random views (1×100 magnification) to calculate TUNEL ratio.

### Western blotting assay

Cells and tumor tissues were incubated with RIPA lysis buffer (Biyuntian). Thirty micrograms of lysates per lane were separated on 10% sodium dodecyl sulfate (SDS)-polyacrylamide gel electrophoresis (PAGE) gels, and transferred to polyvinylidene difluoride (PVDF) membranes (Millipore). After blocking, the blots were incubated with the applied primary and secondary antibodies. The enhanced chemiluminescence (ECL) reagents (GE Healthcare) were added to the blots to detect the targeted protein bands. Quantification of the band intensity was performed with Quantity One 4.6.2 software (Bio-Rad, Hercules, CA).

### Reactive oxygen species (ROS) assay

As described^19^, the ROS levels were tested by using the carboxy-H2DCFDA dye. Following treatment, cells were stained with carboxy-H2-DCFDA (10 μM) for 30 min under the dark. The DCF fluorescence was measured under 485 nm excitation and 525 nm emission using the Fluorescence machine (Thermo Scientific, Shanghai, China).

### Glutathione content assay

Reduced glutathione (GSH) is one key scavenger of ROS, and its ratio with oxidized disulfide form glutathione (GSSG) can be used as a quantitative indicator of oxidative stress intensity^20^. Following treatment, cells were lysed. The ratio of reduced to oxidized glutathione (GSH/GSSG) was measured using the GSH/GSSG assay kit (Beyotime).

### Sphingosine kinase 1 (SphK1) activity assay

For each treatment, 200 μg lysates were incubated with 25 μM D-erythro-sphingosine in 0.1% Triton X-100, 2 mM ATP, and [^γ-32^P] ATP for 30 min at 37 °C^21^, stopped by adding 20 μL of HCl, plus 800 μL of chloroform/methanol/HCl (100:200:1, v/v). Afterwards, chloroform and KCl (250 μL each) were added, and centrifugation was performed to separate layer phases. The organic layer was dried and resuspended in chloroform/methanol/HCl^21^. Lipids were resolved on silica TLC plates in 1-butanol/acetic acid/water^21^. Labeled sphingosine-1-phosphate (S1P) spots were visualized by autoradiography and quantified by scraping and counting in a scintillation counter. SphK1 activity was evaluated as pmol/h/g protein.

### Ceramide content assay

The cellular ceramide level was analyzed by the protocol reported early^22^, tested as fmol by nmol of phospholipids.

### Mitochondrial depolarization

As described^23^ following stress-induced mitochondrial depolarization, JC-1 dye shall aggregate in mitochondria, forming green monomers^24^. RCC were seeded onto the 24-well tissue-culturing plates (1 × 10^4^ cells per well). Following SC66 treatment cells were incubated with JC-1 (5 μg/mL) for 30 min, washed and tested immediately under a fluorescence spectrofluorometer at 550 nm.

### AKT1 short hairpin RNA (shRNA)

AKT1 shRNA lentivirus (sc-29195V, Santa Cruz Biotech, 10 μL/mL medium) was added to 786-O cells for 24 h. Stable cells were selected by puromycin (5.0 μg/mL) for another 10 days. Expression of AKT1 in the stable cells was determined by Western blotting assay.

### AKT knockout

The small guide RNA (sgRNA) targeting human *AKT1* (Target DNA sequence, 5’-TCACGTTGGTCCACATCCTG) was inserted into the lenti-CRISPR-GFP-puro plasmid^25^. The construct was then transfected to 786-O cells by Lipofectamine 2000. FACS was performed to sort the GFP-positive 786-O cells. The resulting single cells were further cultured in the selection medium with puromycin (5 μg/mL) for 10 days. AKT1 knockout in stable cells was verified by Western blotting assay.

### Xenograft model

Female CB-17 severe combined immunodeficiency disease (SCID) mice, 4–5 week old, 17–18 g, were provided by the Animal Center of Soochow University (Suzhou, China). 786-O cells (6 × 10^6^ per mouse, in 200 μL DMEM/Matrigel, no serum) were subcutaneously (s.c.) injected into flanks. After three week, the xenografts, close to 100 mm^3^, were established (“Day-0”). Ten mice per group were treated once daily by gavage with either vehicle control or SC66 (10 or 25 mg/kg body weight) for 24 consecutive days. Every six days, the mice body weights and bi-dimensional tumor measurements^18^ were recorded. The animal protocol was approved by the Institutional Animal Care and Use Committee (IACUC) of Soochow University and Ethics Review Board of Soochow University (Suzhou, China).

### Statistical analysis

The investigators were blinded to the group allocation during all experiments. Results were expressed as the mean ± standard deviation (SD). Statistical analysis among different groups was performed via one-way analysis of variance (ANOVA) with Scheffe’s test using SPSS20.0 software (SPSS Inc., Chicago, IL). The two-tailed unpaired *T* test (Excel 2007) was applied to test the significance of the difference between two treatment groups. *P* values of <0.05 were considered statistically significant.

## Results

### SC66 inhibits RCC cell progression in vitro

**Fig. 1:**
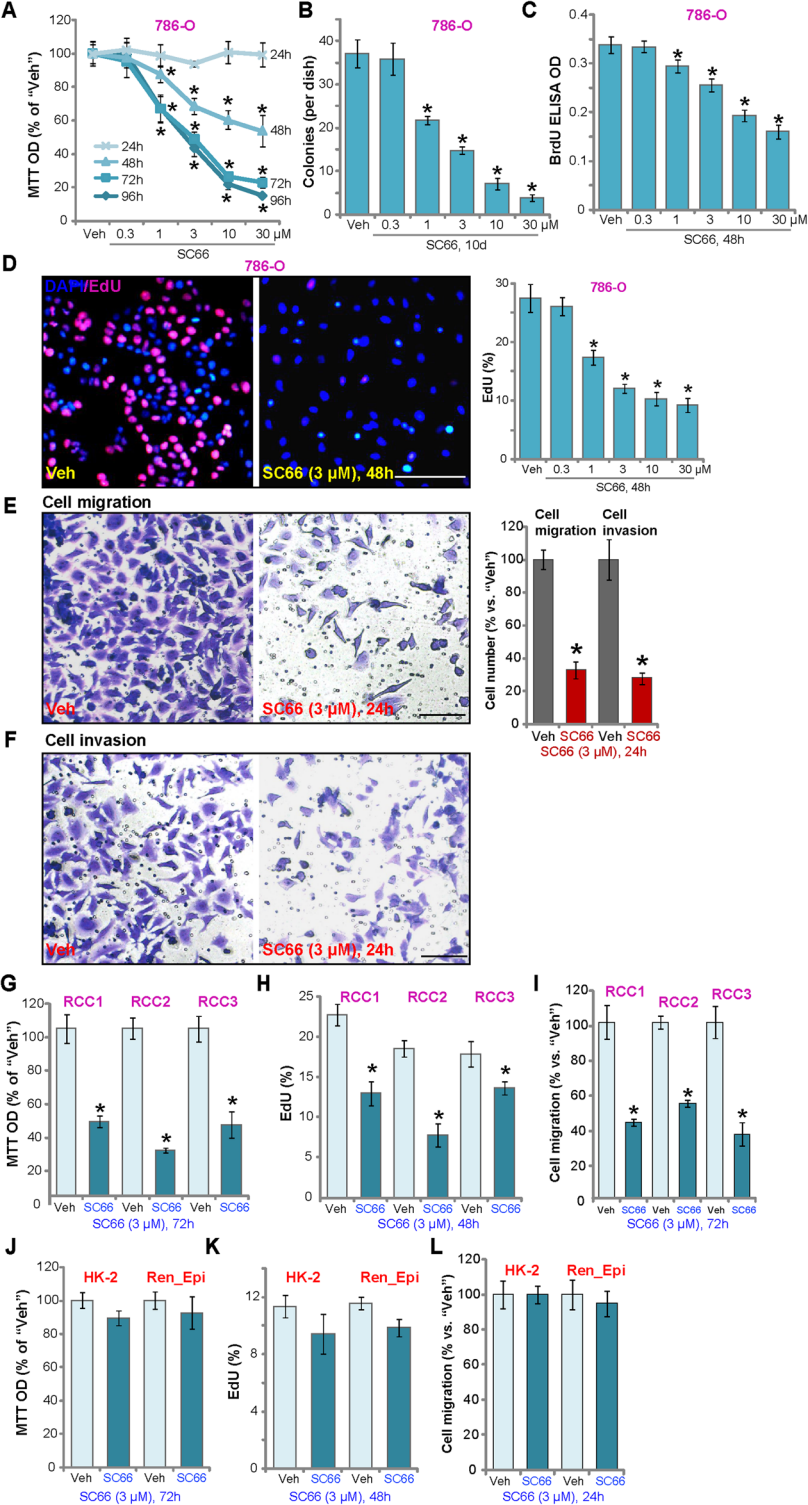
SC66 inhibits RCC cell progression in vitro. 786-O RCC cells (**a**–**f**), primary human RCC cells (“RCC1/RCC2/RCC3”, **g**–**i**), or HK-2 tubular epithelial cells (**j**–**l**), the primary human renal epithelial cells (“Ren_Epi”) (**j**–**l**) were treated with indicated concentration of SC66, cells were further cultured for applied time periods, cell functions, including cell survival, proliferation, migration and invasion were tested by the appropriate assays. For each assay, *n* = 5. Data were expressed as the mean ± standard deviation (S.D.). *P < 0.05 vs. DMSO (0.1%) vehicle (“Veh”, same for all Figures). In this figure, experiments were repeated three times, and similar results were obtained each time. Bar = 100 μm (**d**–**f**, **h**).

To study the mechanism of SC66 cytotoxicity cultured human RCC786-O cells^8–10^ were treated with different concentrations of SC66. The MTT assay of cell viability demonstrated that SC66 dose-dependently reduced the viability of 786-O cells (Fig. 1a), in a time-dependent manner that required at least 48 h to exert a significant effect (Fig. 1a). The IC-50 of SC66 was close to 3 μM at 72 h and 96 h (Fig. 1a), and soft agar colony studies demonstrated that SC66 (1–30 μM) significantly decreased the number of viable786-O cell colonies (Fig. 1b). Examining 786-O cell proliferation, both BrdU ELISA and EdU staining confirmed that SC66 inhibited nuclear BrdU incorporation (Fig. 1c) and EdU incorporation (Fig. 1d) in a dose dependent manner. Measuring cell migration and invasion, Transwell and Matrigel Transwell assays, respectively, demonstrated that SC66 (3 μM, 24 h) potently inhibited 786-O cell migration (Fig. 1e) and invasion (Fig. 1f) in vitro. Similar results were obtained with the A498 human RCC cell line^8,9^, where SC66 (3 μM, 48/72 h) decreased cell viability (Fig. S1A) and proliferation (Fig. S1B), and inhibited A498 cell migration and invasion (Fig. S1C, D).

In the primary human RCC cells, derived from three RCC patients (“RCC1/RCC2/RCC3”), SC66 potently reduced viability (Fig. 1g) and decreased proliferation (Fig. 1h). Transwell results, Fig. 1i, showed that SC66 (3 μM, 24 h) significantly decreased the number of migrated RCC cells. In contrast, immortalized HK-2 tubular epithelial cells^26,27^ and the primary human renal epithelial cells (“Ren-Epi”, from Dr. Hu^28^) were resistant to SC66, showing no significant effect on viability, proliferation or migration (Fig. 1j–l).

### SC66 provokes apoptosis activation in RCC cells

**Fig. 2:**
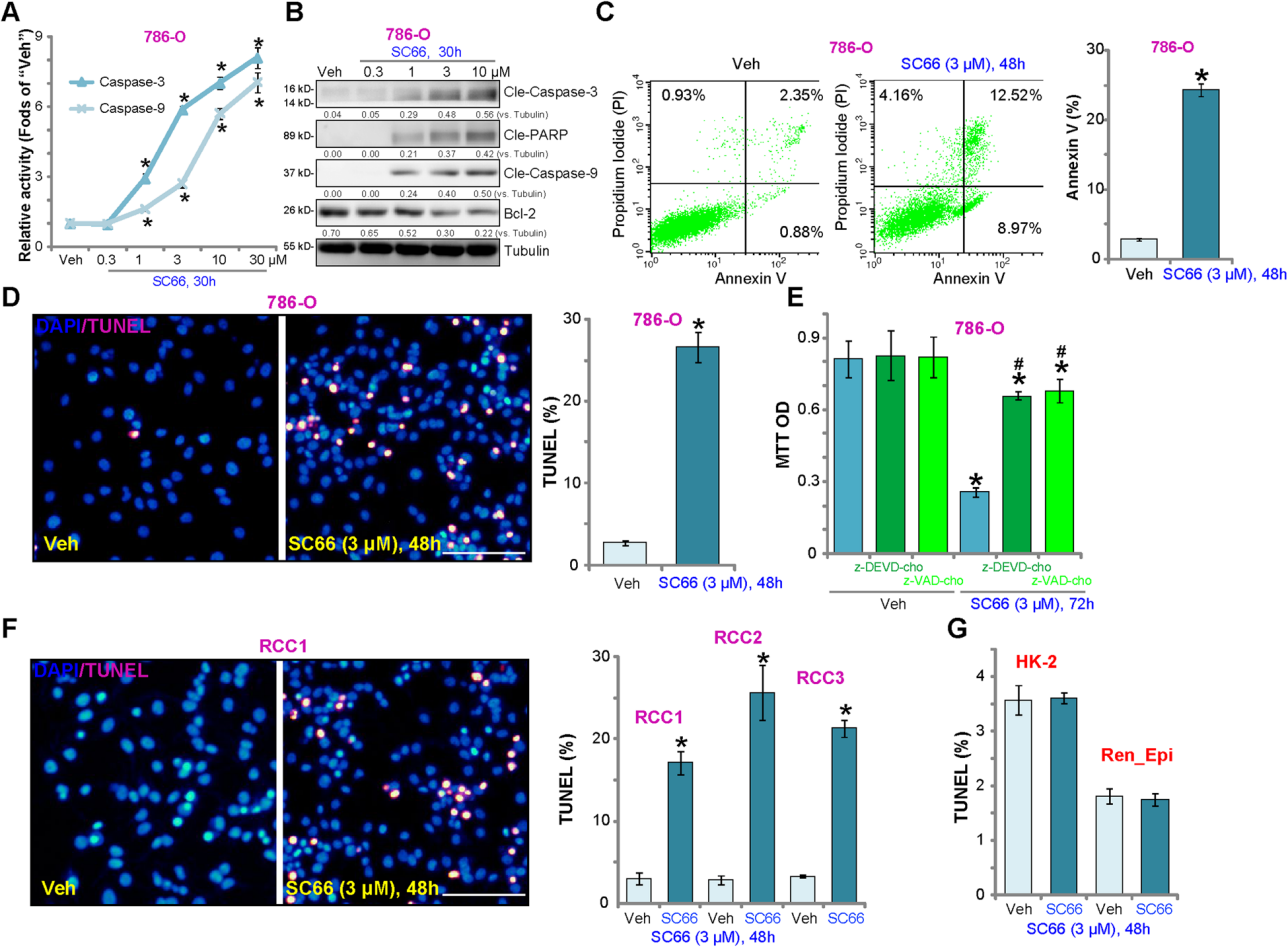
SC66 provokes significant apoptosis activation in RCC cells. 786-O RCC cells (**a**–**e**), primary human RCC cells (“RCC1/RCC2/RCC3”, **f**), HK-2 tubular epithelial cells (**g**), or the primary human renal epithelial cells (“Ren_Epi”) (**g**) were treated with indicated concentration of SC66, cells were further cultured for applied time periods, caspase-3/-9 activities (**a**), expression of apoptosis-associated proteins (**b**) and cell apoptosis (**c**–**d**, **f**, **g**) were tested by the mentioned assays. For **e**, 786-O cells were co-treated with 50 μM of the caspase-3 inhibitor z-DEVD-cho or the pan caspase inhibitor z-VAD-cho, and cell viability was tested by MTT assay. Expression of listed proteins were quantified, normalize to Tubulin (**b**). For each assay, *n* = 5. Data were expressed as the mean ± standard deviation (S.D.). *P < 0.05 vs. “Veh” group. ^#^*P* < 0.05 vs. SC66 treatment only (**e**). In this figure, experiments were repeated three times, and similar results were obtained each time. Bar = 100 μm (**d** and **f**).

Using the previously described methods^8–10,15^, we tested the effect of SC66 on cell apoptosis. As shown, SC66 dose-dependently increased the activities of caspase-3 and caspase-9 in 786-O cells (Fig. 2a). Analyzing apoptosis-associated proteins, SC66 (1–10 μM) induced cleavage of caspase-3, caspase-9, and PARP [poly (ADP-ribose) polymerase], and downregulatedBcl-2 (Fig. 2b). Annexin V FACS assay results demonstrated that SC66(3 μM) mainly induced apoptosis (Annexin V^+/+^) in 786-O cells (Fig. 2c). Furthermore, the percentage of cells with positive nuclear TUNEL staining was significantly increased following SC66 treatment (Fig. 2d). Significantly, co-treatment of the caspase-3 inhibitor z-DEVD-cho or the pan caspase inhibitor z-VAD-cho largely attenuated the SC66 (3 μM, 72 h)-induced viability reduction in 786-O cells (Fig. 2e). Similar results were observed in the A498 cell line (Fig. S1E–S1I). In primary human RCC cells (“RCC1/RCC2/RCC3”), treatment with SC66 induced apoptosis activation, as evidenced by a significant increase in nuclear TUNEL staining (Fig. 2f). In line with the above results showing that immortalized HK-2 tubular and primary renal epithelial cells are resistant to SC66, no significant apoptosis was detected (Fig. 2g).

### SC66 inhibits AKT-mTOR activation in RCC cells

**Fig. 3:**
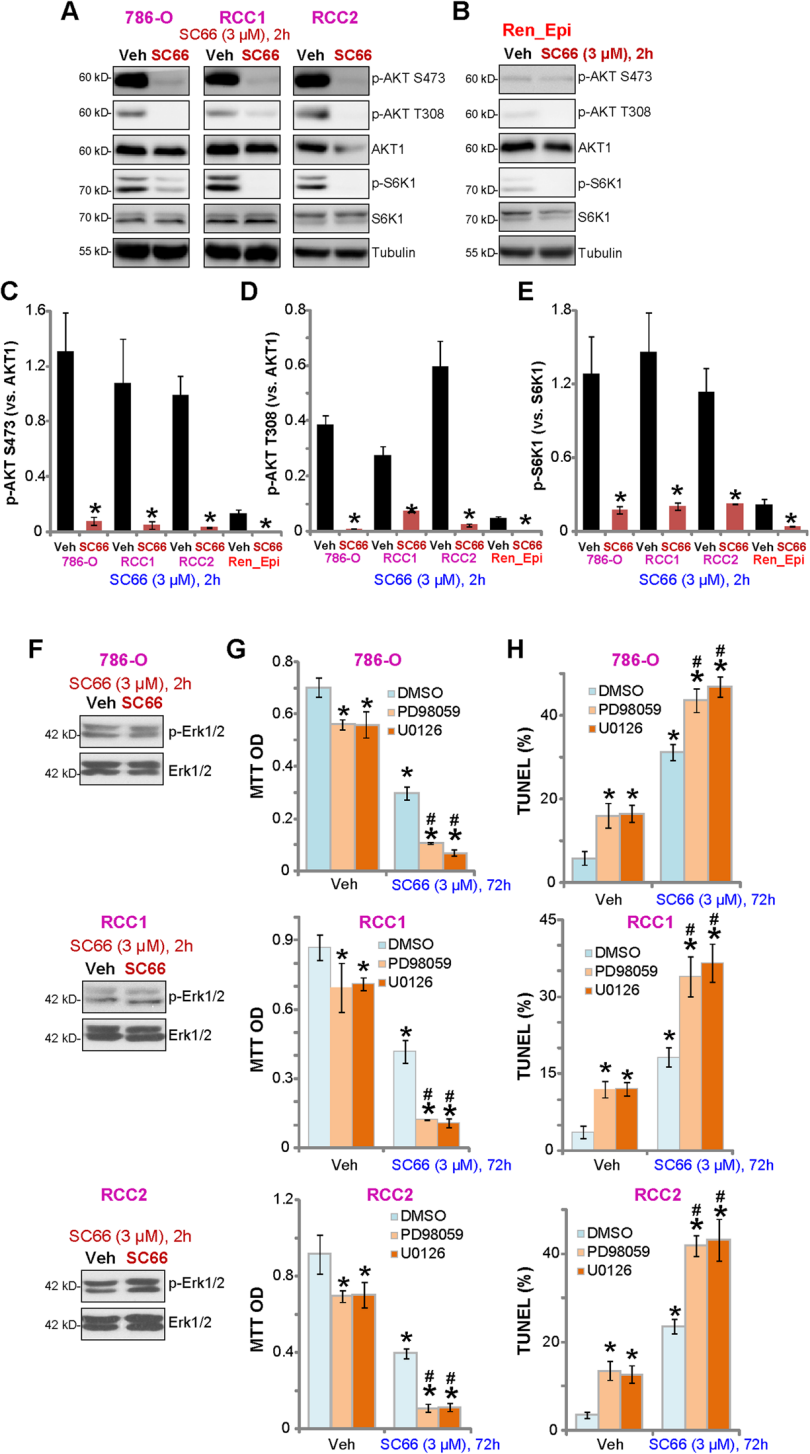
SC66 inhibits AKT-mTOR activation in RCC cells. 786-O cells, the primary human RCC cells (“RCC1/2”), or the renal epithelial cells (“Ren_Epi”) were treated with SC66 (3 μM) for 2 h, expression of listed proteins in total cell lysates were tested by Western blotting assay (**a**, **b**, and **f**).The quantified results integrating five sets of blotting data were presented (**c**–**e**). 786-O cells or RCC1/2 primary cells were treated with SC66 (3 μM), together with or without Erk inhibitor PD98059/U0126 (each at 5 μM), cells were further cultured for 72 h, and cell viability and apoptosis tested by MTT (**g**) and nuclear TUNEL staining (**h**) assays, respectively. Data were expressed as the mean ± standard deviation (S.D.). “DMSO” stands for 0.1% DMSO (**g**, **h**). *P < 0.05 vs. “Veh” group. ^#^*P* < 0.05 vs^.^ “DMSO” plus SC66 treatment (**g**, **h**). In this figure, experiments were repeated three times, and similar results were obtained each time.

As SC66 is reported to inhibit Akt in hepatocellular carcinoma cells^11^^,12,29^, we tested AKT and mTOR signaling in SC66-treated RCC cells. Western blot results demonstrated that phosphorylation of AKT (at both Ser-473 and Thr-308) and S6K1 (at Ser-389) were inhibited by SC66 (3 μM, 2 h) in both 786-O and primary RCC cells (“RCC1/RCC2”) (Fig. 3a). These results confirm that that SC66 acts to block AKT, mTORC1 (indicated by p-S6K1^30^^,31^) and mTORC2 (indicated by p-AKT at Ser 473^30^^,31^) in RCC cells (Fig. 3a). Total AKT1 protein level was also decreased by SC66 treatment in RCC cells (Fig. 3a), possibly due to ubiquitin-mediated degradation^11^. Quantified results, integrating five sets of repeated blotting data in 786-O and primary RCC cells, show that SC66-induced significant AKT-mTORC1/2 inhibition (Fig. 3c–e). Basal AKT-mTORC1/2 activity was significantly lower in the primary renal epithelial cells (Fig. 3b–e), possibly explaining the ineffectiveness of this compound on normal epithelial cells (Figs. 1 and 2).

As Erk signaling plays a role in RCC oncogenesis^13,32–34^, we examinedp-Erk1/2 at Thr202/Tyr204, finding that it was unchanged followingSC66 (3 μM, 2 h) treatment in 786-O and primary RCC cells (Fig. 3f). However, co-treatment with the Erk inhibitors, PD98059 and U0126, significantly potentiated SC66-induced viability reduction (Fig. 3g) and apoptosis (Fig. 3h). Treatment with the Erk1/2 inhibitors alone induced minor but significant cytotoxicity in the tested RCC cells (Fig. 3g, h). These results suggest that Erk inhibition could sensitize SC66-induced cytotoxicity in human RCC cells.

### SC66 induces oxidative stress, SphK1 inhibition, and JNK activation in RCC cells

**Fig. 4:**
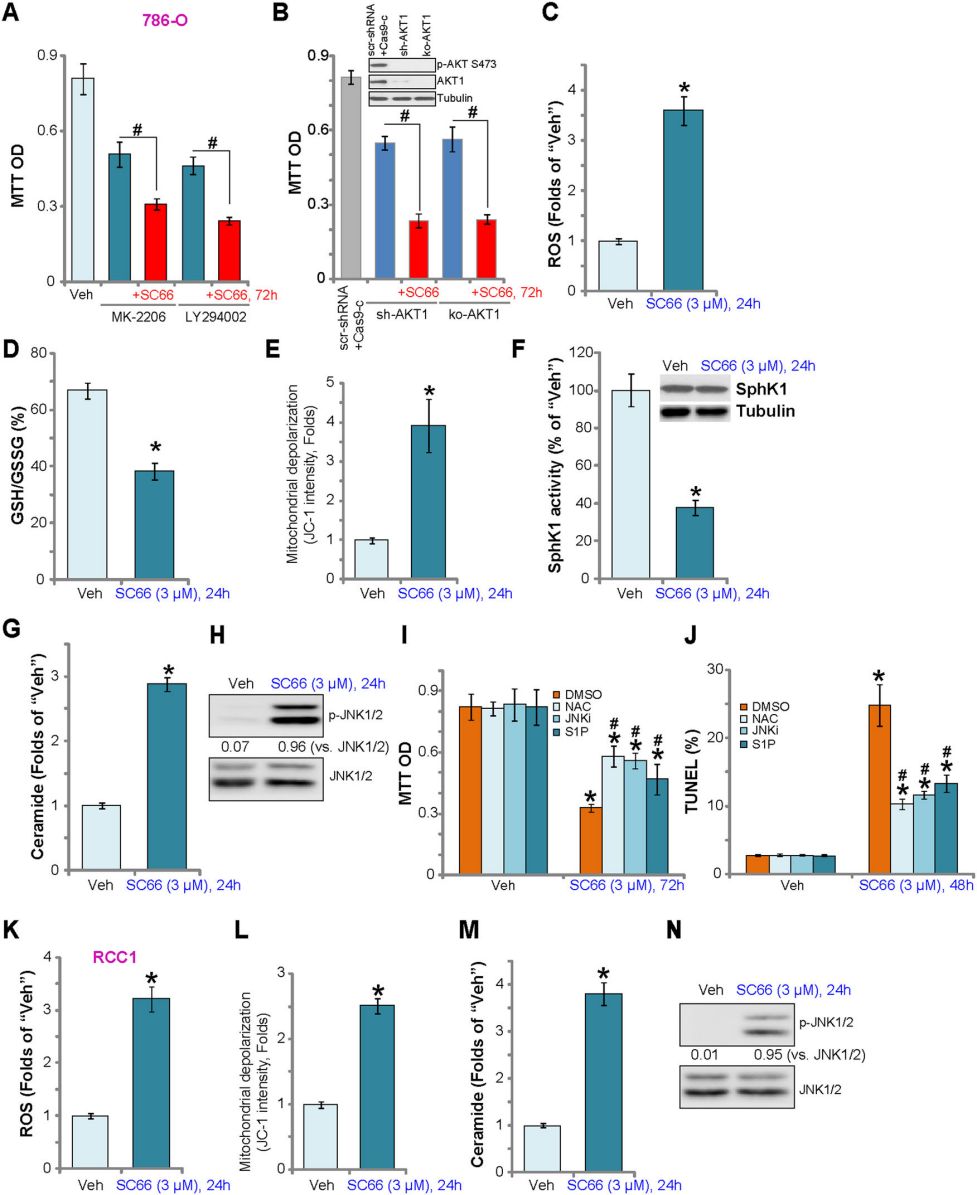
SC66 induces oxidative stress, SphK1 inhibition, and JNK activation in RCC cells. 786-O cells were treated with MK-2206, LY294002 or plus SC66 (all at 3 μM), cells were further cultured, and cell viability was tested by MTT assay (**a**, 72 h). Stable 786-O cells with AKT1 shRNA (“sh-AKT1”) or CRISPR/Cas9 AKT1-KO construct (“ko-AKT1”), as well as the control cells with scramble control shRNA and Cas9 empty plasmid (“scr-shRNA+Cas9-c”), were tested by Western blotting assay of AKT expression (**b**, the upper panel), cells were treated with/without SC66 (3 μM) for 72 h, cell viability was tested (**b**, the lower panel). 786-O cells or the primary human RCC cells (“RCC1”) were treated with SC66 (3 μM) for indicated time periods, ROS production (**c** and **k**), GSH/GSSG ratio (**d**), mitochondrial depolarization (**e** and **l**), SphK1 expression and activity (**f**), as well as the ceramide contents (**g** and **m**) and p-/t-JNK expression (**h** and **n**) were tested by the appropriate assays. 786-O cells were pretreated for 30 min with NAC (400 μM), JNKi (10 μM), or S1P (10 μM), followed by SC66 (3 μM) treatment for 48 and 72 h, cell viability and apoptosis were tested by MTT assay (**h**) and TUNEL staining assay (**i**), respectively. Phosphorylated JNK1/2 was normalized to total JNK1/2 (**h** and **n**). For each assay, *n* = 5. Data were expressed as the mean ± standard deviation (S.D.). *P < 0.05 vs. “Veh” group. ^#^*P* < 0.05 vs^.^ SC66 treatment only (**i**, **j**). In this figure, experiments were repeated three times, and similar results were obtained each time.

To examine whether SC66-induced cytotoxicity is primarily via Akt inhibition, we compared SC66 efficacy with known AKT inhibitors, including the AKT specific inhibitor MK-2206^35^ and the PI3K-AKT-mTOR pan inhibitor LY294002^36^. MTT assay results (Fig. 4a**)** demonstrate that SC66 was more potent than MK-2206 and LY294002 in inhibiting786-O cell viability. Significantly, SC66 further reduced the viability of 786-O cells pretreated with MK-2206 and LY294002 (Fig. 4a), suggesting that SC66 effects are not limited to AKT inhibition. To confirm an AKT-independent mechanism of SC66 cytotoxicity in 786-O cells, shRNA and CRISPR/Cas9 methods were applied to silence and knockout AKT1, respectively (Fig. 4b, the upper panel). Results show that SC66 was cytotoxic in AKT1-silenced/-KO cells (Fig. 4b), indicating AKT-independent mechanisms of killing 786-O cells.

Exploring possible AKT-independent mechanisms, we found that SC66 induced oxidative stress in 786-O cells, evidenced by increased ROS production (DCF-DA intensity increase, Fig. 4c) and reduced GSH/GSSG ratio (Fig. 4d). In addition, SC66 increased mitochondrial depolarization, tested by JC-1 green monomers formation (Fig. 4e). Furthermore, sphingosine kinase 1 (SphK1) activity, an enzyme that prevents ceramide accumulation, was inhibited in SC66-treated 786-O cells (Fig. 4f) with a concomitant increase in ceramide levels (Fig. 4g). A consequence of ceramide accumulation is pro-apoptotic JNK activation^37^, and a significant increase of JNK1/2 phosphorylation was detected in SC66-treated cells, confirming JNK activation (Fig. 4h**)**. To examine whether these pathways are involved in the cytotoxic action of SC66, we tested the effects of the ROS scavenger NAC, the JNK inhibitor JNKi, and anti-ceramide sphingolipidsphingosine-1-phosphate (S1P). As shown, SC66-induced viability reduction (Fig. 4i) and apoptosis (Fig. 4j) were inhibited by pretreatment with NAC, JNKi or S1P. In the primary human RCC cells (“RCC1”), SC66 treatment similarly reduced ROS production (Fig. 4k), mitochondrial depolarization (Fig. 4l), ceramide accumulation(Fig. 4m) and JNK activation (Fig. 4n). Thus, AKT-independent mechanisms participated in SC66-induced cytotoxicity in RCC cells.

### SC66 inhibits 786-O xenograft tumor growth in SCID mice

**Fig. 5:**
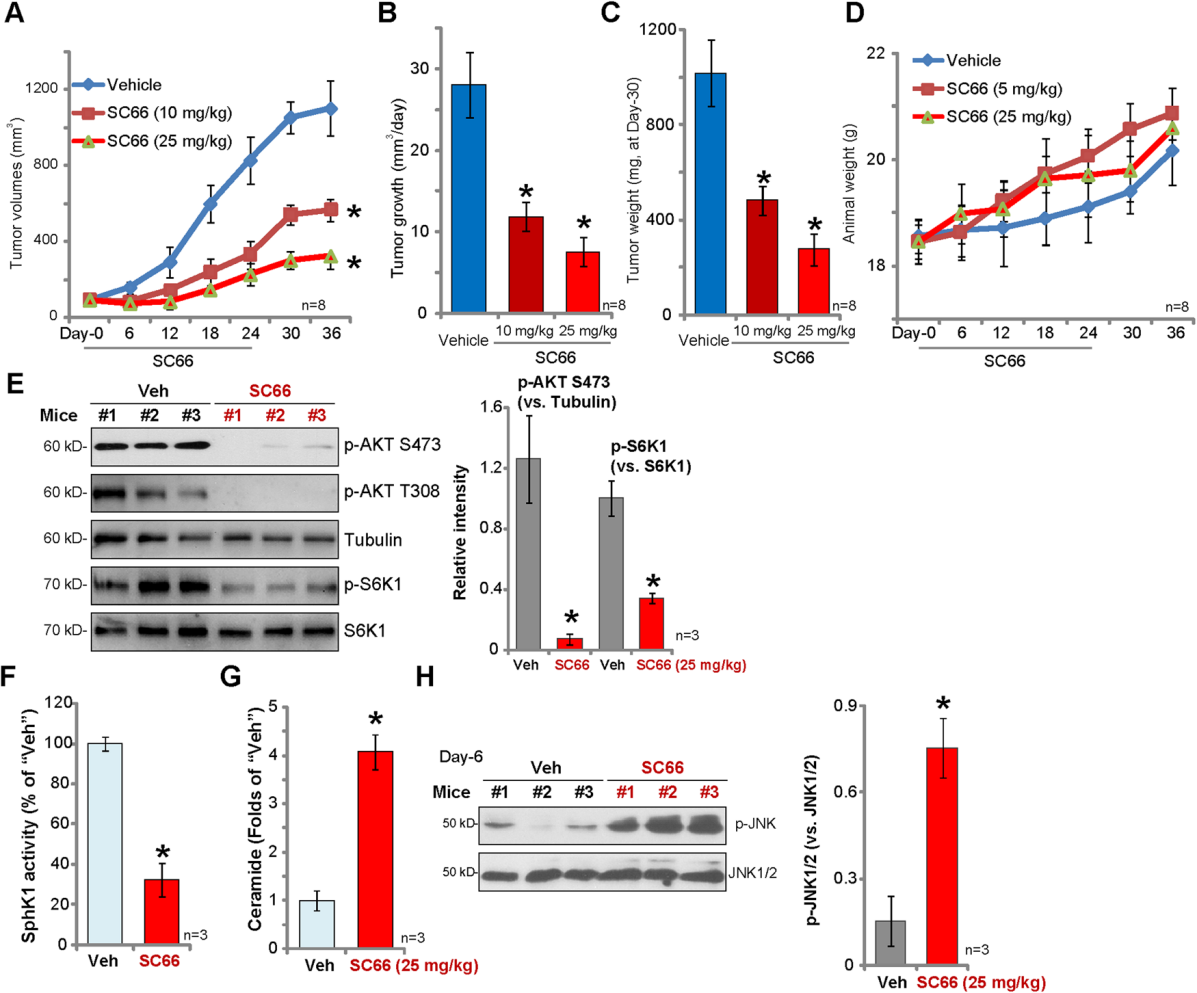
SC66 inhibits 786-O xenograft tumor growth in SCID mice. 786-O tumor-bearing SCID mice (eight mice per group, *n* = 8) were orally administrated with SC66 (10/25 mg/kg body weight, daily, for 24 days) or vehicle control (“Vehicle”), tumor volumes (**a**) and mice body weights (**d**) were recorded every 6 days. Estimated daily tumor growth was calculated as described (**b**). At Day-36, tumors were isolated and weighted (**c**). At treatment Day-6, two hours after SC66 (25 mg/kg) or vehicle administration, three 786-O tumors (*n* = 3) of each group (total six tumors) were isolated; Expression of listed proteins in tumor lysates was tested by Western blotting assays (**e** and **h**); The relative SphK1 activity (**f**) and ceramide contents (**g**) in tumor lysates were tested as well. The quantified results integrating all three sets of blotting data were presented (**e** and **h**). Data were expressed as the mean ± standard deviation (S.D.). *P < 0.05 vs. “Vehicle” group.

We tested the potential effect of SC66 in vivo using the previously-described 786-O xenograft tumor model^8,9^. 786-O cells were *s.c*. injected into the flanks of SCID mice and xenografts established within three weeks when tumors were around 100 mm^3^ (“Day-0”). We found that oral administration of SC66, at 10 and 25 mg/kg body weight, significantly inhibited tumor volume(Fig. 5a), and that daily tumor growth was significantly inhibited (Fig. 5b). At Day-36, tumors from all three groups were isolated and weighted individually.SC66-treated 786-O tumors weighted significantly less than the vehicle control tumors (Fig. 5c), while mouse body weights were not significantly different between the three groups (Fig. 5d). At treatment Day-6, two hours after SC66 (25 mg/kg) or vehicle administration, three 786-O tumors from each group (total six tumors) were isolated. Analyzing signaling changes, AKT-S6K phosphorylation was significantly inhibited in SC66-treated tumor lysates (Fig. 5e), confirming AKT-mTOR inhibition. In line with the in vitro findings, SC66 treatment decreased SphK1 activity (Fig. 5f), increased ceramide levels (Fig. 5g), and increased JNK activation (Fig. 5h).

## Discussion

Our study shows that SC66 inhibited cell viability, proliferation, migration and invasion in established (786-O and A498 lines) and primary human RCC cells.SC66 was found to inhibit AKT-mTORC1/2 activation and induce significant apoptosis in RCC cells. In contrast, this AKT inhibitor was non-cytotoxic to HK-2 epithelial cells and primary human renal epithelial cells with low basal AKT-mTORC1/2 activation. In vivo, SC66 oral administration, at well-tolerated doses, potently inhibited subcutaneous 786-O xenograft growth in SCID mice.

mTORC1 inhibitors are approved by FDA for the treatment of advanced RCC patients after failure of either sunitinib or sorafenib^2,3^. However, the use of mTORC1 inhibitors can have several drawbacks. First, the mTORC1 inhibitors, rapamycin and its analogs (“rapalogs”), only indirectly inhibit mTORC1^38,39^. Second, mTORC1 inhibition can induce feedback activation of the PI3K-AKT and ERK-MAPK oncogenic pathways^38–40^. Third, rapalogs are unable to directly inhibit mTORC2, the latter being equally as important as mTORC1 in RCC progression. We have previously shown that WYE-687, a mTORC1/2 dual inhibitor, inhibited RCC cell growth with greater efficiency than mTORC1 inhibitors^9^. Further, the mTORC1/2 inhibitor, AZD2014, exerted more potent anti-RCC cell activity than rapalogs^18^. Similarly, the finding that SC66 can block AKT and mTORC1/2 activation in established and primary RCC cells is an advantage of this compound.

Furthermore, SC66 also exhibits cytotoxic actions independent of AKT1. Here, we show that in RCC cells SC66 induced ROS production, SphK1 inhibition, ceramide accumulation and JNK activation, which does not occur in RCC cells treated with the AKT specific inhibitor MK-2206^35,41^ or in AKT1-silenced/-KO RCC cells. Significantly, the ROS scavenger NAC, the JNK inhibitor and anti-ceramide sphingolipid S1P all mitigated, but did not reverse, SC66-induced cytotoxicity in RCC cells. Importantly, confirming in vitro results, SphK1 inhibition, ceramide accumulation and JNK activation were detected in SC66-treated 786-O xenograft tumors. Therefore, SC66 acts through both AKT-dependent and AKT-independent mechanisms to exert more potent anti-RCC activity.

SphK1 is over-expressed and/or hyper-activated in RCC, promoting cancer progression^42,43^. SphK1 phosphorylates sphingosine to form S1P^44,45^, and SphK1 inhibition or silencing induces ceramide accumulation to promote cell apoptosis. Despite the importance of sphingolipid-derived signaling in tumorigenesis, there is a lack of potent and selective inhibitors of SphK. We found that SC66 inhibits SphK1 activation leading to pro-apoptotic ceramide accumulation and JNK activation in vitro and in vivo. Further studies are needed to determine the mechanism by which SC66 inhibits SphK1 in RCC cells.

It has been shown that Erk activation contributes to everolimus-acquired resistance and a poor prognosis in RCC patients^33^. Contrarily, Erk inhibition enhanced the efficacy of everolimus against RCC cells^33^. Yuen et al., found that AZD6244, an Erk inhibitor, at low doses augmented the antitumor activity of sorafenib^34^. In this study, we show that inhibition of Erk by PD98059 or U0126 potentiated SC66-induced cytotoxicity and apoptosis in 786-O and primary RCC cells, indicating that Erk activation could be a key resistance mechanism of SC66 in RCC cells.

## Conclusion

In summary, we show that SC66 inhibits RCC cell progression in vitro and in vivo, through AKT-dependent and AKT-independent mechanisms. It should be noted that the findings of in vitro and animal RCC studies could not be directly translated to humans, and thus the efficacy and safety of SC66 need to be further investigated and confirmed.

## Supplementary information

Figure S1

Supplementary Figure legends
