# Supplementary figures and images for: The therapeutic value of SC66 in human renal cell carcinoma cells
Source: Cell Death Dis. 2020 May 11;11(5):353. doi: 10.1038/s41419-020-2566-1 (PMC7214466; doi:10.1038/s41419-020-2566-1)

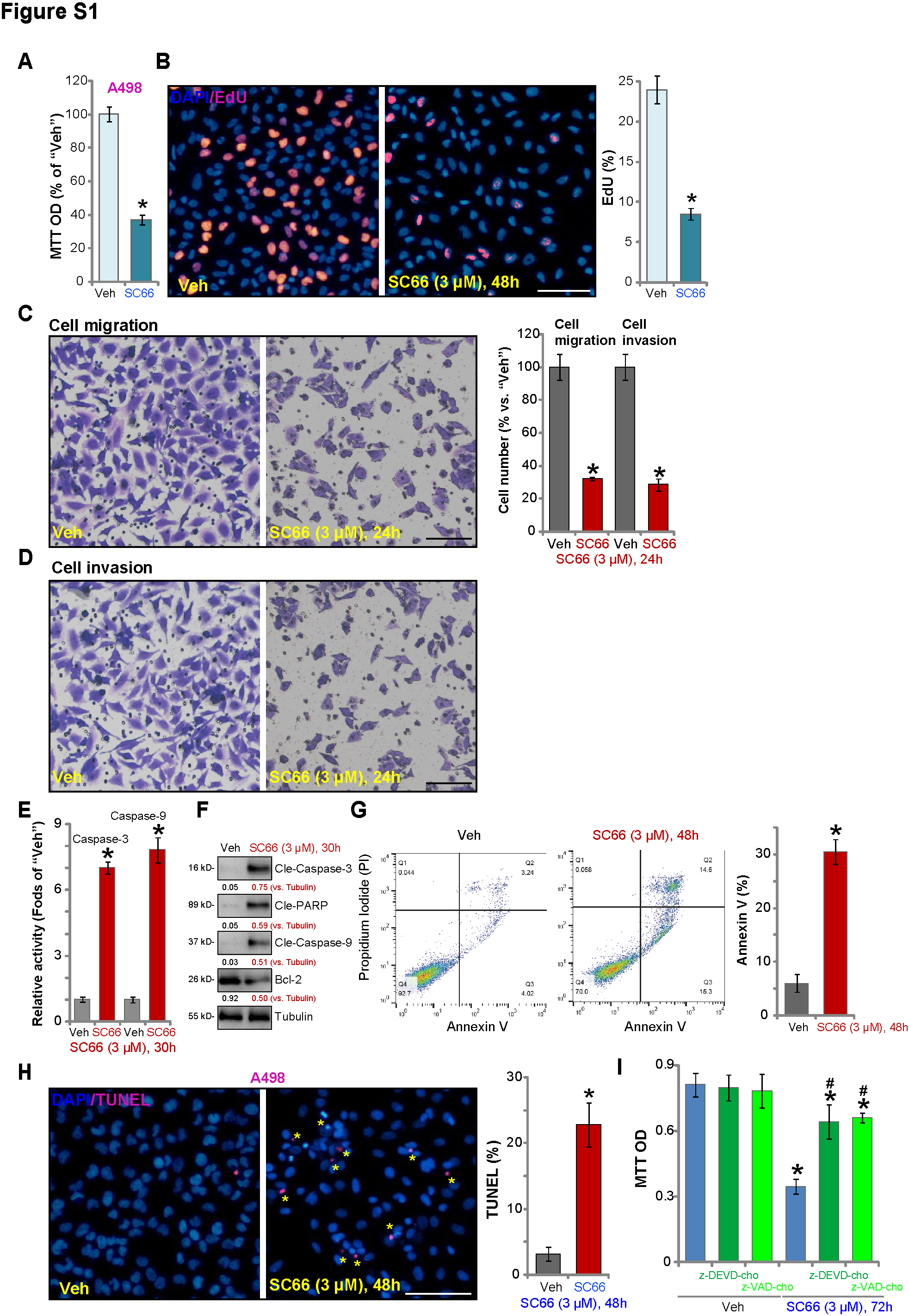

Supplement: Supplementary file 1 — Figure S1 [file 41419_2020_2566_MOESM1_ESM.tif]
