# Supplementary material for: The therapeutic value of SC66 in human renal cell carcinoma cells
Source: Cell Death Dis. 2020 May 11;11(5):353. doi: 10.1038/s41419-020-2566-1 (PMC7214466; doi:10.1038/s41419-020-2566-1)
Supplement: Supplementary file 2 — Supplementary Figure legends [file 41419_2020_2566_MOESM2_ESM.docx]

**Figure S1.**A498 cells were treated with SC66 (3 μM) and further cultured for applied time periods, cell viability (**A**), proliferation (**B**), migration (**C**) and invasion (**D**) were tested by the appropriate assays; Caspase-3/-9 activities (**E**), expression of apoptosis-associated proteins (**F**) and cell apoptosis (**G** and **H**) were tested as well. For (**I**), A498 cells were co-treated with 50 μM of z-DEVD-cho or z-VAD-cho, and cell viability tested by MTT assay after 72h of SC66 treatment. Expression of listed proteins were quantified, normalize to Tubulin (**F**). For each assay, n=5. Data were expressed as the mean ± standard deviation (S.D.). **P*< 0.05 vs. “Veh” group. ^#^*P*< 0.05 vs. SC66 treatment only (**I**). Experiments were repeated three times, and similar results were obtained each time. Bar = 100 μm (**B**-**D**, **H**).
